# Amyloid‐β in Alzheimer's disease: Structure, toxicity, distribution, treatment, and prospects

**DOI:** 10.1002/ibra.12155

**Published:** 2024-05-23

**Authors:** Yifan Yu, Shilong Yu, Giuseppe Battaglia, Xiaohe Tian

**Affiliations:** ^1^ Institute for Bioengineering of Catalunya (IBEC) The Barcelona Institute of Science and Technology (BIST), Barcelona (Spain), Carrer Baldiri I Reixac Barcelona Spain; ^2^ Catalan Institution for Research and Advanced Studies (ICREA) Barcelona Spain; ^3^ Department of Radiology and Huaxi MR Research Center (HMRRC), Functional and Molecular Imaging Key Laboratory of Sichuan Province, West China Hospital Sichuan University Chengdu China

**Keywords:** Aβ, Alzheimer's disease, anti‐Aβ drugs, multivalency, nanodrugs

## Abstract

Amyloid‐β (Aβ) is a pivotal biomarker in Alzheimer's disease (AD), attracting considerable attention from numerous researchers. There is uncertainty regarding whether clearing Aβ is beneficial or harmful to cognitive function. This question has been a central topic of research, especially given the lack of success in developing Aβ‐targeted drugs for AD. However, with the Food and Drug Administration's approval of Lecanemab as the first anti‐Aβ medication in July 2023, there is a significant shift in perspective on the potential of Aβ as a therapeutic target for AD. In light of this advancement, this review aims to illustrate and consolidate the molecular structural attributes and pathological ramifications of Aβ. Furthermore, it elucidates the determinants influencing its expression levels while delineating the gamut of extant Aβ‐targeted pharmacotherapies that have been subjected to clinical or preclinical evaluation. Subsequently, a comprehensive analysis is presented, dissecting the research landscape of Aβ across the domains above, culminating in the presentation of informed perspectives. Concluding reflections contemplate the supplementary advantages conferred by nanoparticle constructs, conceptualized within the framework of multivalent theory, within the milieu of AD diagnosis and therapeutic intervention, supplementing conventional modalities.

## Graphical abstract

The formation, deposition, and toxicity of amyloid‐β (Aβ) are discussed in this article. Aβ is produced after amyloid precursor protein cleavage and chronologically aggregates into monomer, oligomer, fibril, and plaque. Geographical deposition of Aβ will cause synaptic dysfunction, mitochondrial dysfunction, inflammation, or neurodegeneration depending on the balance of the clearance system and deposition, the structural properties of Aβ, the activities of microglia and astrocytes, and the mutation of APP, Presenin‐1/2, and APOE, which are also the targets of BACE1, anti‐Aβ antibodies, and multivalent nanoparticles.

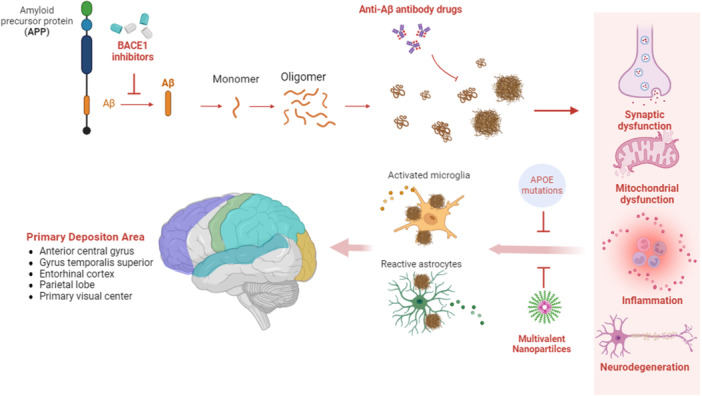

## 1 INTRODUCTION

The amyloid‐β (Aβ) hypothesis, alternatively referred to as the Aβ cascade theory, is central to understanding Alzheimer's disease (AD). In 1991,^1^, ^2^, ^3^ the Aβ theory proposed that amyloid plaque, or its main component, Aβ, was the direct cause of progressive neurodegeneration. Subsequently, advancements in molecular biology, pathophysiology, and clinical research have clarified the role of Aβ, and the theory of Aβ has also been elaborated in different ways. Thus, Aβ has become the most important biomarker for AD diagnosis and the target of many AD therapies. For the latest theoretical developments, Karran's work serves as a pertinent reference for Aβ hypothesis.^4^, ^5^

Research on the relationship between Aβ and AD has been conducted extensively, specifically focusing on inhibitory and excitatory neurons in neuronal networks, microglia, astrocytes, oligodendrocytes, and so forth, to elucidate how many neural‐related factors promote the progression of AD and how the initial benign response eventually becomes a chronic response, leading to irreversible cerebral imbalances. Despite numerous breakthroughs in Aβ, many therapeutic translations have encountered failures because of side effects and insufficiency to remove Aβ, which lead to skepticism regarding the potential benefits of Aβ clearance. However, the landscape shifted significantly with the Food and Drug Administration (FDA) approval of Lecanemab in July 2023, marking a milestone in AD drug development.^6^

Accordingly, this comprehensive review first summarizes the molecular structure characteristics, pathological effects, and factors affecting the expression level of Aβ, then describes the existing Aβ drugs that have been subjected to clinical trials or animal tests, and finally discusses the research landscape of Aβ. We hope to highlight the following questions: (1) What is the molecular structure of Aβ? What kind of characteristics does it possess? (2) What are the mechanisms underlying Aβ‐induced neurotoxicity? (3) What factors affect the expression level of Aβ in vivo, and which of these factors can be modulated artificially? (4) Do Aβ drugs verified in animal models have potential transformation value? (5) What is the current status of Aβ drugs for which clinical trials have been completed? To help readers obtain meaningful information on Aβ, we have briefly summarized highlights of previous reviews ^4^, ^5^, ^7^, ^8^, ^9^, ^10^, ^11^, ^12^, ^13^, ^14^, ^15^, ^16^, ^17^ (Table 1).

**Table 1:** Review mentioned in the article.

Year	Title	Brief summary and highlights	References
2022	Apolipoprotein E and Alzheimer's Disease: Findings, Hypotheses, and Potential Mechanisms.	(a) ApoE4 promotes the production and fibrillization of Aβ and impairs pathways involved in its degradation/clearance, causing an accumulation of toxic Aβ species and the formation of amyloid plaques. (b) ApoE4 exacerbates the neuroinflammatory response, impairs the ability of astrocytes to maintain synapses, and causes microglia to increase phagocytosis of neurons and decrease removal of toxic proteins, including Aβ and tau aggregates. (c) Due to the unique apoE4‐domain interaction, using a small‐molecule structure corrector to disrupt the domain interaction would cause apoE4 to resemble apoE3 both structurally and functionally. (d) One potential approach based on the gain‐of‐toxic‐function effects of apoE4 is the reduction of apoE4.	[17]
2022	The amyloid hypothesis in Alzheimer's disease: new insights from new therapeutics	(a) The amyloid therapeutic hypothesis and clinical trials of anti‐Aβ drugs. (b) Introducing the approaches targeting soluble Aβ and amyloid plaques. (c) Discussion of the threshold and therapeutic hypothesis for amyloid‐lowering drugs and anti‐Aβ antibodies, and finally a comparison.	[4]
2021	Neuroinflammation and microglial activation in Alzheimer disease: where do we go from here?	(a) Microglia are the primary players in neuroinflammation. (b) Microglia show diverse phenotypes and have multifaceted interactions with Aβ as well as neuronal circuits. (c) The diverse influences of microglia on the progression of AD depend on the stage of disease, individual susceptibility, and state of microglial priming.	[7]
2020	Cerebral amyloid angiopathy and Alzheimer disease—one peptide, two pathways	(a) The Aβ deposits as cerebral amyloid angiopathy (CAA) along vessel walls and causes vascular dysfunction to reduce perivascular Aβ clearance. (b) Early termination of Aβ, missense mutations, and co‐deposited proteins will promote vascular Ab deposition over parenchymal deposition. (c) The imaging abnormalities and side effects in the trials of anti‐Aβ immunotherapy may result from vascular Ab deposition.	[8]
2019	Apolipoprotein E and Alzheimer's disease: pathobiology and targeting strategies	Describes the role of APOE subtypes in the pathogenesis and progression of AD, including the Aβ pathway, tau, and microglia, based on the results of existing basic experiments and clinical trials.	[9]
2019	A critical appraisal of amyloid‐β‐targeting therapies for Alzheimer's disease	(a) Drugs designed to decrease Aβ production, antagonize Aβ aggregation, or increase brain Aβ clearance did not yield clinical benefits. (b) Aβ accumulation represents an epiphenomenon rather than a cause of AD.	[5]
2018	The glymphatic pathway in neurological disorders	The relationship between AQP‐based clearance pathologies of endolymphatic and meningeal lymphatic systems and neuropathologic diseases was introduced.	[10]
2017	Apolipoprotein E and Alzheimer's disease: the influence of apolipoprotein E on amyloid‐β and other amyloidogenic proteins.	Discusses the role of apoE in the biophysical properties and metabolism of the Aβ peptide, the principal component of amyloid plaques and CAA.	[11]
2017	Amyloid precursor protein and endosomal–lysosomal dysfunction in Alzheimer's disease: inseparable partners in a multifactorial disease	(a) The origins of endosomal–lysosomal network (ELN) dysfunction and Aβ genesis closely overlap. (b) Genes that promote Aβ genesis in AD have primary effects on ELN function. (c) Growing evidence implicates AD gene‐driven ELN disruptions as not only the antecedent pathobiology that underlies Aβ genesis but also as an essential partner with APP.	[12]
2017	Towards an understanding of amyloid‐β oligomers: characterization, toxicity mechanisms, and inhibitors	(a) Soluble and structured oligomers are toxic. (b) Aβ oligomers can trigger toxicity in cellular systems by disruption of membranes and dysfunction of organelles, and spread further by cell‐to‐cell transmission. (c) Natural products, synthetic molecules, and protein domains have been developed to inhibit Aβ aggregation, including oligomerization.	[13]
2017	A systemic view of Alzheimer's disease ‐ insights from amyloid‐β metabolism beyond the brain	The relationship between systemic abnormalities and Aβ metabolism not only affects brain dysfunction but also has a relationship with the overproduction and lower clearance of Aβ in turn.	[14]
2015	Clearance systems in the brain implications for Alzheimer's disease.	The effect of the brain clearance system on the progression of AD was described for the first time.	[15]
2014	BACE1 (β‐secretase) inhibitors for the treatment of Alzheimer's disease.	The pharmacological mechanism, pharmacokinetic characteristics, side effects, and clinical research status of BACE1 in the treatment of AD were described in detail for the first time.	[16]

## 2 MOLECULAR STRUCTURE OF AΒ

**Figure 1:**
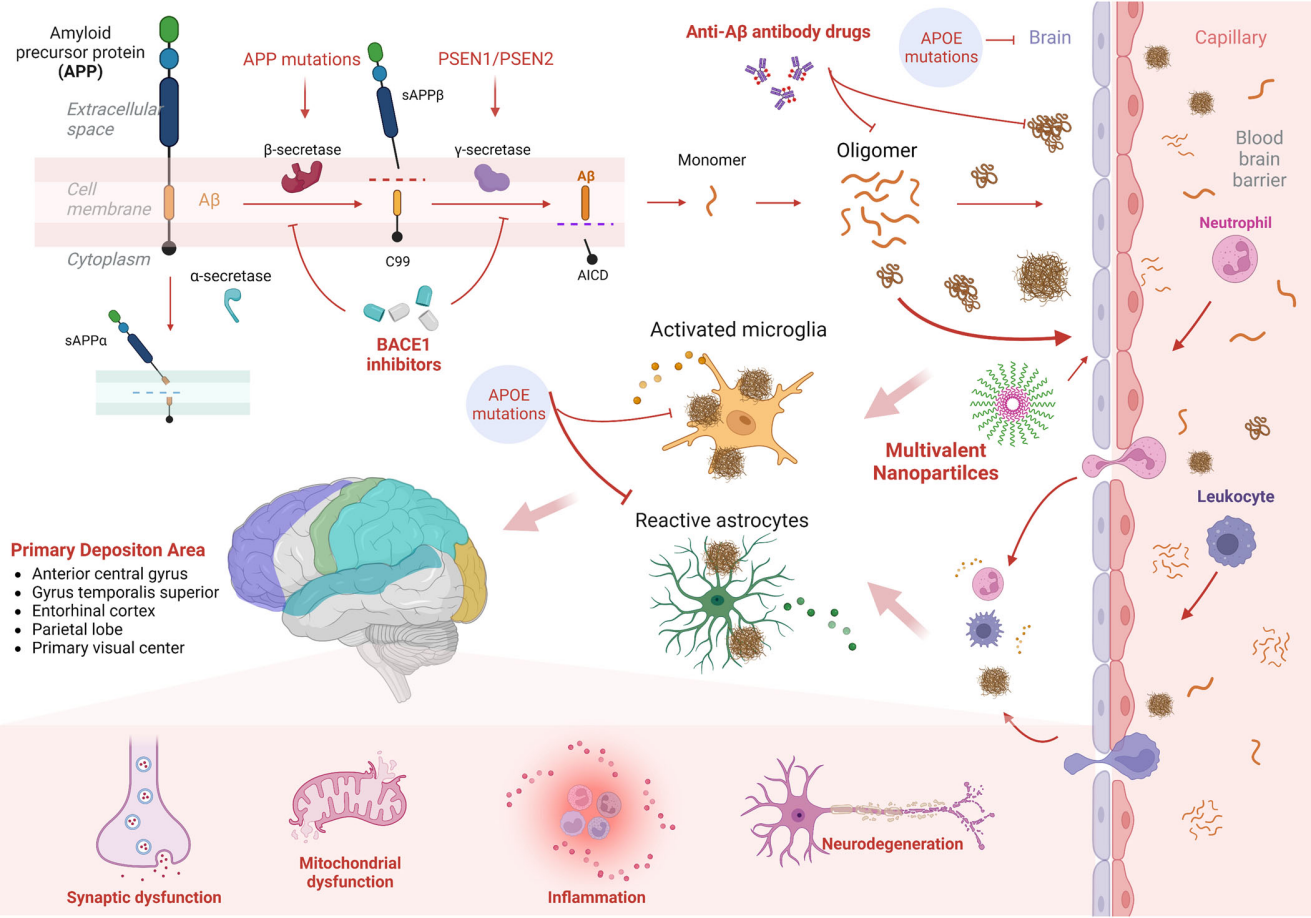
Formation, circulation, deposition, and toxicity of amyloid‐β (Aβ). The amyloid precursor protein is cleaved by α‐secretase and γ‐secretase or β‐secretase and γ‐secretase, and the latter will produce Aβ. Monomer, oligomer, fibril, and plaque are different forms of Aβ in the chronological aggregation period, which will be cleared out of the brain by the clearance system (like the blood–brain barrier clear system) or deposit in the brain. Geographical deposition of Aβ will cause synaptic dysfunction, mitochondrial dysfunction, inflammation, or neurodegeneration depending on the balance of the clearance system and deposition, the structural properties of Aβ, the activities of microglia and astrocytes, and the mutation of APP, Presenin‐1/2, and APOE, which are also the targets of BACE1, anti‐Aβ antibodies, and multivalent nanoparticles. Aβ, amyloid‐β; APP, amyloid precursor protein; APOE, apolipoprotein E; PSEN‐1/2, presenin‐1/2. (Created with BioRender.com). [Color figure can be viewed at wileyonlinelibrary.com]

The Aβ peptide arises from the metabolism of amyloid precursor protein (APP), in which three enzymes are involved, namely, α‐, β‐, and γ‐secretases. α‐secretase cleaves APP's α‐site (C‐terminal), proximate to the cell membrane. β‐Secretase (beta‐site amyloid precursor protein cleaving enzyme 1, BACE1) was identified as a transmembrane aspartic acid protease in 1999,^18^, ^19^ which mainly cleaves APP's β‐site (N‐terminal). γ‐secretase is an enzyme complex composed of presenilin, nicastrin, anterior pharynx defective 1, and presenilin enhancer 2. These three enzymes affect APP cleavage via two distinct pathways: the nonamyloid hydrolytic pathway mediated by α‐ and γ‐secretases and the amyloid hydrolytic pathway mediated by β‐ and γ‐secretases. In the nonamyloid hydrolytic pathway, APP is cleaved by α‐ and γ‐secretases and thus produces the soluble extracellular fragment (sAPPα) with the neuroprotective effect that can prevent Aβ formation and modulate synaptic transmission.^20^ However, in the amyloid hydrolysis pathway, APP will be cleaved by β‐secretase to generate sAPP and cell membrane‐bound fragment (C99), and C99 will subsequently be cleaved by γ‐secretase to generate Aβ (Figure 1).

The Aβ molecules, generated through enzymatic actions of β‐ and γ‐secretases, result in two primary forms: Aβ_1‐40_, composed of 40 amino acids and the most abundant form, and Aβ_1‐42_, consisting of 42 amino acids with insolubility. Once a certain length of Aβ is formed, it will start seeding and aggregating to form oligomers, protofibrils, fibrils, and plaques. The size of Aβ is primarily influenced by its inherent molecular attributes and some protein denaturation during its biosynthesis.^21^ At the same time, the aggregation of Aβ depends on the distribution of residue hydrophobicity.^22^

Regarding the attributes of Aβ, different Aβ_1‐42_ forms may emerge through subtle changes in their respective growth conditions. When the fibril grows from a pre‐formed seed, the morphology and molecular structure show self‐replicating characteristics.^23^ Using new cryo‐electron microscopy with 4.0 Å resolution and solid‐state nuclear magnetic resonance experiments, Gremer et al.^24^ proved that the distribution of N‐terminal and C‐terminal Aβ will affect the growth of Aβ through different binding pathways. From the protein perspective, AD‐associated mutations in presenilin (PSEN) and APP lead to enhanced synthesis of elongated Aβ peptides, since PSEN or APP mutations greatly reduce the activity of γ‐secretase ^25^ and destabilize the E–S complex secretase.^26^

Upon initiation of Aβ aggregation, the hydrophobic regions of Aβ are encapsulated in the Aβ fibrils, while the hydrophilic regions are exposed on the surface. Due to the difference in residue distribution, Aβ_42_ is more likely to aggregate rapidly and form fibrils. In all aggregates, the most toxic forms of Aβ were oligomers rather than monomers and amyloid fibrils.^27^ According to their size, oligomers can be divided into small oligomers composed of dimers or trimers, medium‐sized oligomers in the form of 9mers, and protofibrils. Previous studies have shown that the relative amount of deposition of Aβ oligomers is significantly lower than that of total Aβ, indicating that these soluble oligomers do not accumulate over time but may become undetectable fibrous structures,^28^ which is consistent with a new view that seeding is a rare and slow process.^29^

Studies on oligomers are challenging due to their heterogeneity and metastability. Prior investigations have established that oligomers may disrupt normal synaptic function or impede the generation of new synapses by affecting the function of mitochondria or microglia, leading to the decline of cognition or learning ability.^27^ Subsequent investigations, using a diverse array of techniques, have delved into the details of the assembly and structural properties of oligomers, as described in Lee SJ's research.^13^ However, despite these efforts, the factors driving the conformational transformation into oligomers remain unclear.

## 3 PROPERTIES AND TOXICITY OF AΒ

The underlying mechanism through which Aβ induces cellular dysfunction has remained elusive. Nevertheless, extant studies suggest that it ultimately damages nerves by affecting the integrity and stability of cell membranes.^30^, ^31^ The toxicity of Aβ manifests in direct and indirect ways. Direct toxicity occurs when Aβ accumulates and its inherent chemical properties are activated, directly affecting cell functions. In contrast, indirect toxicity occurs when normal nerve function is damaged due to inflammatory responses, damaging blood vessels, or triggering secondary response (Figure 1).

### 3.1 Prion‐like infectivity

Aβ can be seen as a prion‐like pathological protein whose phenotype depends on the host and pathogen. Intracerebral injections of diluted Aβ‐containing brain extracts from AD patients or APP transgenic mice have been shown to induce brain Aβ synthesis and related pathological changes in a time‐ and dose‐dependent manner.^32^ Similar prion activity was also shown in the brains of APP transgenic mice.^33^ In addition, Aβ seeds also showed prion‐like behavior, and the expression of this behavior depends on the expression of the APP genotype.^34^ In APP(−/−) (APP null mouse) brains, Aβ seeds can exist without the occurrence of amyloidosis, but amyloidosis occurred when Aβ seeds were transferred to the brains of APP(+/+) mice. This finding was later also observed in humans, with Purro et al.^35^ reporting that there was Aβ deposition in prion‐contaminated human cadaver pituitary‐derived growth hormone, and those who received the treatment with it in childhood would present Aβ pathology. Recently, researchers found iatrogenic AD in recipients of cadaveric pituitary‐derived growth hormone.^36^ Furthermore, this process may be enhanced by microglia, as it can facilitate the proliferation of prion‐like Aβ in both Aβ‐affected^37^ and Aβ‐unaffected^38^ brain tissues.

### 3.2 Direct toxicity

The direct toxicity of Aβ primarily included damage to the mitochondria, endosome–lysosome system, and cell transport mechanisms. The generation and spread of this direct toxicity are related to activity‐regulated cytoskeleton‐associated protein (Arc) protein, paired immunoglobulin‐like receptor B (PirB), and active transcriptional factor 4 (ATF4) protein. The molecular mechanism of mitochondrial toxicity is the extensive alteration of the active site in the crystal structure of Aβ ‐binding alcohol dehydrogenase (ABAD), which prevents the binding of nicotinamide adenine dinucleotide (NAD) in the mitochondria and thereby produces more Aβ.^39^ Furthermore, damage to the endosome–lysosome system was caused by changes in the activity of Rab5.^40^ It should be noted that Kwart and Nixon proposed that a β fragment of APP, rather than Aβ, induced endosomal–lysosomal dysfunction in APP and presenilin‐1 (PS1) mutant individuals.^12^, ^41^ In addition, Aβ disrupted the endocytic trafficking of plasma membrane receptors, damaging glutamatergic neurons and primary rat cortical neurons.^42^ Further studies confirmed that Aβ can inhibit the reuptake of glutamic acid, causing hyperactivity of sensory neurons. The consequential excessive accumulation of glutamate would lead to overproduction of Aβ,^43^ forming an Aβ‐dependent vicious circle.

The immediate early gene Arc was a pivotal factor for Aβ activity‐dependent production. Arc is a postsynaptic protein that recruits endotrophic 2/3 and dynein to early/cycling endosomes that reduce synaptic strength by transporting both Hebbian and non‐Hebbian forms of the α‐amino‐3‐hydroxy‐5‐methyl‐4‐isoxazole‐propionic acid receptor (AMPAR). Arc‐endosomes also transport APP and BACE1 and regulate γ‐secretase trafficking through physical association with PS1.^44^ Meanwhile, PirB of mice and its human homolog leukocyte immunoglobulin‐like receptor B2 (LilrB2), present in the human brain, are receptors for Aβ oligomers and are indispensable for the harmful effects of Aβ oligomers on long‐term potentiation of the hippocampus. In a transgenic model of AD, PirB not only caused memory deficits but also mediated loss of plasticity of synapses in the juvenile visual cortex.^45^

ATF4 protein is the intermediary of AD pathological spread related to neurodegeneration and axonal translation. ATF4 protein and its transcripts are more frequently expressed in axons of AD patients. Local application of Aβ would initiate the axon to synthesize ATF4, resulting in the loss of nerve cells and the pathological spread of Aβ.^46^

### 3.3 Indirect toxicity: Immunity and inflammation

Aβ will indirectly lead to impairment of synaptic function, neuronal cell death, and inhibition of nerve regeneration by inducing the release of Interleukin‐1β (IL‐1β), IL‐6, IL‐18, tumor necrosis factor (TNF), chemokines, prostaglandins, nitric oxide, oxygen free radicals, and so forth.^47^, ^48^ Neuroinflammation is related to the activation of the immune system, and the participating immune cells in the central neural system (CNS) are mainly microglia. Acting as the phagocytic entities of the CNS, microglia, under normal conditions, are responsible for the phagocytosis of redundant neurons, induction of neuronal apoptosis, and synaptic pruning.^49^, ^50^

From the perspective of the pathological distribution of Aβ, microglia and Aβ are closely related spatially. In 1988, the presence of microglia was documented in the hippocampus of AD patients.^51^ Further studies have proved that the reduction of Aβ increased the transcription of microglial genes with the colocalization of microglia and Aβ.^52^ Also, around the few fibrous plaques in apolipoprotein E (APOE) (−/−) mice brains, the decrease of microglial proliferation and neurodegeneration were more obvious, suggesting that APOE played an important role in mediating microglial activation and localized Aβ‐induced impairment of neural processes.^53^ On the other hand, the binding of APOE to the competing receptors of Aβ like lipoprotein‐related protein 1 (LRP1) and low‐density lipoprotein receptor 1 (LDLR) on astrocytes, microglia, and endothelial cells impaired Aβ clearance.^54^

From the perspective of molecular changes, in APP/PS1 mice, systemic inflammation can change the morphology of microglia and impair Aβ clearance,^55^ which may promote AD pathological changes and cognitive decline.^56^ At the same time, the toxic effects of neuroinflammation is also associated with complement, in which, C1q is required for the long‐term enhanced toxic effects of soluble Aβ oligomers on synapses and the hippocampus.^57^ Besides, treatment of microglia with Aβ leads to increased expression of CD40, which in turn induces neuronal damage by increasing TNF‐α, and its effect depends on the CD40–CD40 ligand linkage.^58^ In addition, the depletion of activated B cells by anti‐CD20/B220 antibodies ameliorated Aβ pathology in mice.^59^

Leng Fe et al.^7^ expounded the relationship between microglia and neuroinflammation in detail. Combined with the latest research, we can summarize as follows: (1) Activated microglia show different phenotypes and interact differently with Aβ, tau, and neuronal circuits. (2) Activated microglia may have different effects on the progression of AD depending on the stage of the disease, individual susceptibility, and the priming state of microglia. (3) Microglia may be potentially regulated at different stages of AD to prevent or modify disease progression. However, in the context of neurodegenerative diseases, neuroinflammation is often a chronic process that does not resolve on its own and is considered an important driver of disease.

### 3.4 Indirect toxicity: Vascular factors

It is widely acknowledged that the vascular factors of Aβ are closely associated with the blood–brain barrier (BBB), an internal barrier of cerebral micro‐vessels composed of continuous endothelial cells. Neuroimaging and pathological studies have demonstrated BBB dysfunction in AD, which results in increased BBB permeability, microhemorrhages, decreased glucose transport, decreased Aβ clearance, and increased leukocyte infiltration.^60^

A prospective study of normal older adults highlighted the relationship between vascular risks and cognitive decline since it can be independent of or synergistic with Aβ.^61^ It was found that Aβ stimulates cerebral capillary constriction (rather than arterioles) at the pericyte location, leading to vascular changes in the population with cognitive decline.^62^ The underlying mechanism is relevant to the generation of reactive oxygen species by Aβ with the release of endothelin‐1 and the activation of pericyte endothelin‐associated receptors. Concurrently, deficiency of glucose transporter‐1 in endothelial cells triggers the vascular phenotype shown by the breakdown of the BBB.^63^ This initial cerebrovascular degeneration will accelerate Aβ pathology and reduce Aβ clearance and neuronal activity, leading to progressive neurodegeneration and triggering behavioral deficits.

However, the discernment of a potential association between a cerebrovascular disease (CVD) like cerebral amyloid angiopathy (CAA)^11^ and AD has perpetually engendered debate. Some of the imaging signatures of CVD and Aβ or tau may coexist in similar brain regions but lack the temporal relationship to progress at the individual level, suggesting that these processes occur through independent mechanisms. Altering the course of CVD is not expected to directly affect the accumulation of Aβ and tau and subsequent neurodegeneration and cognitive decline.^64^

### 3.5 Toxic effects with the synergy of tau

Tau and Aβ have similar status in AD pathological markers and have correlations at expression and genetic levels.^65^ Tau and Aβ production patterns are closely linked to a common genetic profile of lipid metabolism, with tau‐specific genetic profiles classified as “axon‐associated” and Aβ profiles classified as “dendritic‐associated.” It was previously found that the reduction of tau had a protective effect on neurotoxicity, and reducing endogenous tau can alleviate the neurodegeneration induced by Aβ.^66^ Aβ oligomers exert neurotoxicity by rapidly inhibiting the axonal transport and reducing the tau level. However, elevated tau can also play a dendritic role through the N‐methyl‐d‐aspartic acid receptor to confer postsynaptic Aβ toxicity, mediating Aβ neurotoxicity.^67^ Under the induction of elevated tau, pyroglutamylated Aβ misfolds to form structurally different low‐*n* oligomers, which can spread through a prion‐like mechanism and are more toxic than Aβ_42/40_.^68^ In addition, not only will tau enhance the pathological manifestations of Aβ but it will also exacerbate the pathological progress of tau and increase the accumulation of tau.^69^ In a study of individuals with normal cognition, it was found that the tau/Aβ positive group showed the most severe cognitive decline over time among all individuals with normal cognition.^70^

Although tau aggravates nerve damage by Aβ,^71^, ^72^ it can also be protective in specific cases. Under normal physiological circumstances, the formation of postsynaptic excitotoxic signaling complexes involving Aβ is regulated by neuronal p38 mitogen. Loss of p38γ exacerbates neuronal circuit aberrations, cognitive deficits, and premature neuronal apoptosis. However, the phosphorylation of specific sites of tau can inhibit the neurotoxicity of Aβ by regulating p38γ.^73^

## 4 FACTORS ASSOCIATED WITH AΒ LEVELS

Since AD is a degenerative neuropathy, changes in Aβ levels in long‐term progress are highly significant. The consensus is that Aβ levels are related to APP and APOE genes and associated with abnormal protein activity, sleep, and environmental changes.

### 4.1 APP and APOE

The phosphorylated Serine/Threonine‐Proline (Ser/Thr‐Pro) motif in the peptide of APP has cis‐ or trans‐conformation, conversion of which is catalyzed by Pin1 proline isomerase. Peptidylprolyl Cis/Trans Isomerase NIMA‐Interacting 1 (Pin1) accelerates the rate of APP isomerization by more than 1000 times, overexpression of Pin1 reduces the secretion of Aβ in cell culture, and knockdown of Pin1 increases its secretion. However, in mice, the knockout of Pin1 increases the processing of amyloid‐derived APP and selectively increases insoluble Aβ_42_, a major toxicant, in the brain in an age‐dependent manner.^74^ Whereas the APP mutation (alanine‐673‐valine‐673 [A673V]) affects APP processing only in the homozygous state, resulting in enhanced Aβ production and amyloid fibril formation in vitro, heterozygous carriers are not affected.^75^ In addition, the APP intracellular domain (AICD) downregulates Wiskott–Aldrich syndrome protein (WASP)‐family verprolin homologous protein 1, limiting Aβ production, which is another potential mechanism to regulate Aβ production.^76^

The mutation of the APP gene is the most important risk factor of Aβ. Transgenic mice with mutated APP gene showed AD‐like behavioral, biochemical, and pathological abnormalities, resulting in a fivefold increase in Aβ_1‐40_ and a 14‐fold increase in Aβ_1‐42_ at 9–10 months of age, which were mainly found in cortical and limbic structures.^77^ Because APP is encoded on chromosome 21,^78^ excessive production of Aβ due to the duplication of chromosome 21 in patients with Down's syndrome will be more obvious.

APOE is an apolipoprotein that can transport lipids and cholesterol to target cells through receptor‐mediated endocytosis. LDLR and LRP 1 are the main metabolic receptors that regulate APOE levels and other effects in the brain.^79^ APOE encompasses three main subtypes, APOE4, APOE3, and APOE2, among which APOE 4 has a damaging effect, and APOE3 and APOE2 have a protective effect. APOE4 participates in the pathogenesis of AD by impairing microglial reactivity, lipid transport, synaptic integrity and plasticity, glucose metabolism, and cerebrovascular integrity and function; some of these effects are independent of Aβ‐related pathways. The ability of APOE secreted by glial cells to stimulate neurons to produce Aβ is as follows: APOE4 > APOE3 > APOE2.^80^

APOE4 carriers have more severe deposition of Aβ, earlier onset, and faster disease progression once the symptomatic phase begins.^11^ In clinically normal older adults, elevated Aβ is only associated with APOE4 and family history.^81^ APOE2 has a protective effect, which can reduce the risk of disease in a dose‐dependent manner.^82^ APOE4 carriers account for 66.7% of AD, and it is a very important therapeutic strategy to treat AD by regulating APOE4 to reduce the Aβ load.^83^ APOE3 variants are also protective, reducing Aβ pathology by reducing APOE4 aggregation and increasing cholesterol efflux.^84^

### 4.2 Protein abnormalities

Cells coexpress different γ‐secretase complexes, including two homologous presenilins (PSENs): PS1 and presenilin‐2 (PS2). The phosphorylation of Serine‐threonine kinase‐3 (GSK3) is important for the stability of PS1, and the deposition of Aβ will be reduced after the level of GSK3 is inhibited.^85^ A unique motif in PS2 directs γ‐secretase to late endosomes/lysosomes, and when PS2 becomes abnormal, more aggregation‐prone Aβ_42_ accumulates in acidic compartments such as lysosomes, further increasing Aβ levels.^86^ Another study also found that defects in autolysosomal acidification induce autophagic accumulation of Aβ in neurons in AD, resulting in senile plaques.^87^

The triggering receptor expressed on myeloid cells 2 (TREM2) is a microglial surface receptor that triggers tyrosine phosphorylation of intracellular proteins. TREM2 senses lipids that can bond with Aβ fibers on lipid membranes, maintaining microglial responses to Aβ.^88^ Mutations like R47H can impair this function of TREM2, resulting in reduced Aβ aggregation of microglia and increased apoptosis, which further leads to the aggregation of Aβ in turn. In addition, TREM2 also drives microglial responses to Aβ in a spleen tyrosine kinase (SYK)‐dependent and ‐independent manner.^89^ The SYK pathway will cooperate with disease‐associated microglia to limit the progression of Aβ^90^, ^91^ and exert neuroprotective effects.^92^ In autosomal dominant AD, the TREM2 response initiates the amyloid cascade immediately following the first pathological changes in Aβ aggregation, prompting Aβ plaque deposition.^93^

### 4.3 Clearance system

Whether the elevated Aβ levels are due to excessive Aβ secretion or impaired Aβ clearance has not been determined. Mawuenyega et al. found that groups with higher Aβ levels did not differ in the degree of Aβ production, but their clearance function of Aβ was impaired.^94^ The clearance of Aβ in the brain is mainly accomplished by a unique perivascular channel formed by astrocytes, also known as the endolymphatic system.^15^, ^95^ Rasmussen and Tarasoff‐Conway et al. described the clearance system of Aβ in detail.^10^, ^15^ In subsequent verification studies, it was also found that impaired meningeal lymphatic drainage can exacerbate microglial inflammation, aggravate Aβ deposition, increase microglial proliferation, and lead to neurovascular dysfunction and behavioral deficits in AD patients.^96^

### 4.4 Sleep

The effects of sleep on Aβ levels were mainly related to sleep deprivation at night and increased wakefulness during the day. The dynamics of Aβ are influenced by changes in orexin and sleep cycles, with levels increasing during sleep deprivation and decreasing with dual orexin receptor antagonists.^97^ Sleep deprivation increased Aβ levels by 25%–30% during the night compared to the controls.^98^ Increased waking time increases neuronal activity and inhibits lymphatic system function, reducing the clearance of Aβ and tau, thereby increasing the deposition of Aβ in the brain.^99^ In older adults without dementia, excessive daytime sleepiness (EDS) is associated with increased Aβ deposition.^100^

Taken together, shorter and longer sleep durations will aggravate the deposition of Aβ because the circadian pattern of Aβ is regulated by the production and clearance mechanisms in the sleep–wake cycle. Aβ deposition may impair normal circadian patterns and, thus, both production and clearance mechanisms.^101^ In addition, sleep deprivation also leads to symptoms other than a greater Aβ burden, such as more severe depressive symptoms, higher body mass index, and cognitive decline,^102^ which may be important for the design of future secondary prevention trials.

### 4.5 Synergy of tau

Although previously we emphasized that the neurotoxicity of Aβ is closely related to tau, in terms of the concentration changes, the changes of Aβ and tau did not appear in parallel. For example, recent studies have shown that hypoxia can change tau and Aβ levels to varying degrees.^103^ Examination of tau levels in patients 24 h after cardiac arrest shows that phosphorylated‐tau (p‐tau) increases rapidly in the short term, suggesting that p‐tau is secreted rapidly in the interstitial fluid after hypoxic–ischemic brain injury rather than continuously as NFL or total‐tau (t‐tau), while Aβ42 and Aβ40 concentrations increased over time in most patients but only weakly.

However, although there is no parallel relationship between Aβ and tau, we can still determine their mutual influence. Several factors, such as sex, will influence the relationship between Aβ and tau.^104^ Only in individuals with positive astrocyte reactivity (Ast+) was Aβ associated with elevated plasma p‐tau. In cognitively unimpaired (CU) patients, a functional relationship between the AD‐like pattern of tau entanglement accumulation and Aβ was found only in astrocyte reactivity (Ast+) individuals.^105^ When a sensitive biomarker glial fibrillary protein (GFAP) was used to trace reactive astrocytes and Aβ plasma levels, it was found that GFAP levels were positively correlated with tau pathology only in individuals with Aβ pathology,^106^ which further illustrated the close relationship between Aβ and tau.

## 5 DISTRIBUTION OF AΒ

We not only need to pay attention to changes in Aβ levels but also the accumulation degree and deposition area of Aβ because the accumulation of Aβ is more pathogenic than elevated levels of Aβ.^107^

### 5.1 Detection of Aβ in the brain

Pittsburgh Compound‐B positron emission tomography (PiB‐PET) can diagnose AD with strong specificity (especially in the medial basal and lateral temporal lobe cortex regions) and can be differentiated from other clinical memory impairment diseases. Sensitivity was 96.8% (95% confidence interval [CI], 92.0%–99.1%) and specificity was 87.9% (95% CI, 81.9%–92.4%) in distinguishing AD dementia from all non‐AD neurodegenerative diseases. Among controls with normal cognition, MCI patients, AD dementia patients, and non‐AD neurodegenerative disease patients, the proportions of Aβ‐positive patients were 26.3%, 65.9%, 100%, and 23.8%, respectively.^108^ CU patients aged 65‐85 years with higher initial Aβ PET levels showed higher concentrations of flortaucipir in the temporal region than those with lower Aβ levels.^109^ Regarding prognosis, individuals with baseline cerebrospinal fluid (CSF) Aβ_42_ levels in the lower half of the reference range were more likely to develop subsequent Aβ positivity.^110^

In addition to PET and CSF detection, blood samples have also begun to be used to predict the Aβ content in the brain. A notable study has clinically demonstrated that the levels of Aβ in the blood can effectively correlate with Aβ levels in the brain, as measured by PiB‐PET scans, thus providing a noninvasive means to track AD.^111^ A novel Elecsys immunoassay that can detect the level of plasma Aβ automatically can improve the accuracy of prescreening Aβ in clinical AD trials with the combination of blood APOE detection, which can be used to reduce costs and the number of PET/lumbar punctures.^112^ Based on blood testing techniques, noninvasive biomarkers, such as plasma NFL, have also been beneficial for predicting neurodegeneration in AD and monitoring the efficacy of disease‐modifying drug trials.^113^

### 5.2 Distribution in the brain of AD patients

The deposition of Aβ in the brain of AD patients shows different characteristics according to the genotype, age, and disease progression period of the patient. Elevated levels of total Aβ_40_ and Aβ_42_ were first observed in five neocortical regions (precentral gyrus, superior temporal gyrus, entorhinal cortex, parietal lobe, and primary visual cortex) in the early stages of dementia, which were strongly associated with cognitive decline.^114^ Then, it was observed that Aβ deposition also occurs in other cerebral areas. Recent studies agree that Aβ deposition areas include primary cortices, medial structures, and temporal regions.^115^ According to the time sequence of Aβ deposition, the early region of Aβ accumulation included the precuneus, posterior cingulate, isthmus cingulate, insulin, and medial and lateral orbitofrontal cortices, the late region included the lingual, peri calcarine, paracentral, precentral, and postcentral cortices, and the intermediate region included the remaining regions of the brain with increased accumulation rates.^116^

Besides, genes can also affect the deposition and distribution of Aβ in some ways. Most commonly, polymorphisms in the promoter region of the APOE gene affect the amount of Aβ in AD brains—one‐third of mild to severe AD APOE4 noncarrier Aβ deposits in the cerebral cortex.^117^, ^118^ Children with autosomal dominant AD (ADAD) have increased functional connectivity between the posterior cingulate cortex and the medial temporal lobe region, increased gray matter volume in the temporal region, and abnormal plasma Aβ_1‐42_ levels, but the extent to which the underlying brain changes are neurodegenerative or developmental remains to be determined.^119^ It should be noted that the degree of Aβ deposition is related to the polymorphism of the APOE gene promoter region but not the APOE genotype.^120^

It is generally believed that the deposition of Aβ in different brain regions may correspond to nerve damage in different brain regions. However, whether Aβ deposition leads to nerve damage or whether nerve damage leads to Aβ deposition has always been controversial. For example, a previous study on lysosomal acidification disorders^87^ suggested that nerve damage preceded Aβ deposition, but the synaptotoxicity of Aβ also suggested that Aβ can cause nerve damage.

In 1998, Lippa et al. observed that in patients with PS‐1 mutations, Aβ_42_ deposition preceded other changes in AD.^121^ Then, Yau et al. also demonstrated that the appearance of Aβ preceded the occurrence of neurodegeneration and cognitive decline.^122^ In a longitudinal study of 174 individuals, the researchers found that the more advanced the AD preclinical stage, the more Aβ was deposited longitudinally. Still, this elevated Aβ level alone may not accurately represent the ongoing neurodegenerative process. It was also pointed out that suspected non‐Alzheimer's disease pathophysiology (SNAP) patients seem most likely to have the inherent individual variability in brain structure or comorbid pathological features.^123^ On also considering three mutation carriers (PS1, PS2, and APP), it was found that there were significant temporal and spatial differences between the appearance of pathological markers and changes in brain metabolic function: the precuneus was the first cortical region to show a between‐group difference: 22.2 years earlier than the expected onset of Aβ accumulation, 18.8 years earlier than the anticipated onset of hypometabolism, and 13.0 years earlier than the scheduled onset of cortical thinning.^124^ This suggests that the distribution of Aβ has a certain spatiotemporal specificity, which is related to disease progression and gene mutations.

### 5.3 Distribution in the CSF of AD patients

The spatiotemporal specificity of Aβ is also shown in CSF. Abnormalities of Aβ_42_ in CSF, PET Aβ/tau/brain atrophy, and impairment of brain metabolism and episodic memory appear 25, 15, and 10 years earlier than the expected symptoms of AD, respectively.^125^ In Down syndrome patients, AD has a long preclinical period. During this period, the expression of AD biomarkers in the plasma and CSF is changed,^126^ and this change has a predictable sequence. CSF Aβ1‐42/1‐40 and plasma NFL changes in Down syndrome patients occurred in the third decade and amyloid PET uptake occurred in the fourth decade.^127^ Another study found that although the distribution of PiB–PET signals in the cortex and striatum varied among PSEN1, PSEN2, and APP variants, their CSF Aβ_42_ levels showed similar progression.^128^ This implies that Aβ levels measured by CSF and PET are not simply interchangeable, as they may reflect different Aβ‐driven pathobiological processes.

However, it is undeniable that there is a good correlation between CSF Aβ and cerebral Aβ. Currently, PET is mainly used to measure the Aβ levels in the brain, while the enzyme‐linked immunosorbent assay or mass spectrometry (MS) is primarily used to measure the Aβ content in CSF. Janelidze et al. compared 8 Aβ detection methods in two independent cohorts and showed that certain MS‐based methods outperformed most plasma Aβ_42/40_ immunoassays in detecting brain Aβ pathology.^129^ However, they also improved some immunoassays, making CSF detection of AD more feasible and effective.^130^

## 6 CLINICAL TREATMENT

In this part, we describe the overall landscape of therapeutic drugs of Aβ and point out limitations and future directions of current research. Currently, research and development of therapeutic drugs are mainly focused on enhancing the clearance of Aβ plaque, inhibiting intermediate enzymes, or blocking Aβ precursors or receptors to reduce the concentration or toxic reactions of Aβ and then eliminate neurotoxic effects in the brain. Active immunity is a common means of disease prevention or treatment that induces immune response by injection of agents, thereby enabling the body to secrete immune factors, which improve resistance to pathological reactions and finally clear pathological markers and inhibit pathological reactions. Conversely, passive immunity involves the injection of immune substances synthesized in vitro, such as immunoglobulin, so that the immune substance can target the pathology, induce the immune response, clear the pathology, and neutralize the possible pathogenic factors. Targeting the pivotal enzymes involved in Aβ formation, β‐secretase inhibitors and γ‐secretase inhibitors interfere with its normal function, impeding Aβ production and subsequently attenuating pathological responses.

### 6.1 Immunotherapy

For immunotherapy, in a study carried out in 1999, Schenk et al.^131^ prevented the formation of Aβ in young and old animals by immunizing them with Aβ_42_. Their research elucidated varying mechanisms of Aβ immunization in mice with AD of different ages: in young animals, astrocyte proliferation in the brain was decelerated, while in aged animals, the degree and progression of AD‐like neuropathy were diminished. This marked the advent of immunotherapy in the clinical domain. The existing immune antibodies are mainly human monoclonal or polyclonal antibodies. A few representative immunological drugs are as follows:

AN‐1792^132^: It significantly reduces Aβ deposition by triggering T‐cell‐dependent immunity. However, recent results did not show any clinical benefit, and even caused meningitis.

Vanutide^133^: The original use of this drug was to relieve the side effects of AN‐1792, but the expected clinical benefits ultimately did not occur.

CAD106^134^, ^135^, ^136^, ^137^: It reduced Aβ in a dose‐dependent manner, mainly targeting oligomers and monomers, and did not increase cerebral microbleeds and induce inflammatory reactions. No clinical benefits were achieved either.

Lecanemab (BAN2401)^6^: This is an FDA‐approved monoclonal antibody drug targeting soluble Aβ protofibrils selectively, which improved Aβ deposition in recent trials. Aβ levels were reduced in treated patients with early AD, and cognition and daily living activities improved. However, there were side effects of amyloid‐associated cerebral edema or hydrocephalus (mostly asymptomatic).

Aducanumab^138^, ^139^, ^140^: As a human‐derived recombinant immune globulin G 1 (IgG1) that targets soluble Aβ aggregates and insoluble fibrils and recognizes Aβ amino‐terminal residues 3–7, it successfully clears Aβ in a time‐ and dose‐dependent manner. The main safety and tolerability findings were amyloid‐related imaging abnormalities.

Donanemab^141^: As an antibody against deposited Aβ, it could improve composite scores for cognition and activities of daily living in patients with early AD, but amyloid‐associated cerebral edema or hydrocephalus (mostly asymptomatic) also occurred.

Solanezumab^142^, ^143^: This is a human IgG1 monoclonal antibody, which targets the central region of Aβ and recognizes soluble monomeric Aβ; it could increase the Aβ levels in plasma and CSF in a dose‐dependent manner. However, no expected clinical benefits were achieved in phases I, II, and III, and adverse cerebral edema or effusion lesions were observed on magnetic resonance imaging.

Gantenerumab^142^: As a humanized recombinant IgG1 monoclonal antibody, it could combine with the amino acid end of the Aβ central region and mainly targeted oligomers and fibrils. In clinical phases I, II, and III, it successfully reduced Aβ in a dose‐dependent manner. However, no expected clinical benefit was achieved.

Crenezumab^144^: This is a human IgG4 monoclonal antibody that can bind to aggregated Aβ pentameric oligomers and fibril 16mers. Although it can inhibit Aβ aggregation without inflammatory response, vasogenic edema, and cerebral microbleeds, it did not affect Aβ deposition either.

Bapineuzumab^145^, ^146^: It targets the N‐terminal region of Aβ, but failed to achieve its primary outcome measure.

### 6.2 β/γ secretase inhibitors

In 2001, Luo et al. carried out research on β‐secretase inhibitors and γ‐secretase inhibitors and found that unlike previous mice with α‐secretase knockout, mice with knockout of BACE1 not only survived but also had normal phenotypes.^147^ Although some studies also showed that knockout of BACE1 would lead to some side effects, such as seizure,^148^ memory impairment,^149^ and increased risk of schizophrenic behavior,^150^ they provided a reliable animal model for research of BACE1 therapy. So far, the mechanism of action of BACE1 has not been clarified, but it is indeed an ideal way to inhibit the early formation of Aβ. Ohno et al.^151^ found that memory deficits in AD animals were rescued after genetically inhibiting the activity of this enzyme, which proved the therapeutic potential of BACE1 inhibitors. BACE1 inhibitors mainly include peptide, nonpeptide, natural products with BACE1 inhibitory activity, and nonpeptide inhibitors from computational methods.^16^ Some representative BACE1 and γ‐secretase inhibitors are as follows:

Verubecestat^152^, ^153^: This is a BACE1 inhibitor that could alleviate the accumulation of Aβ at the highest dose of 40 mg without inducing cerebral microbleeds, but without any cognitive and behavioral improvements. Furthermore, its dose overloading resulted in rapid nonprogressive reductions in whole brain and hippocampal volume and even cognitive deterioration.

Lanabecestat^144^: This is a BACE1 inhibitor that was found to reduce Aβ in CSF in a dose‐dependent manner, but it did not meet any clinical expectations. Side effects include worsened psychiatric adverse events, weight loss, and hair color changes. Thus, the trail of this drug had been terminated after an invalid analysis.

Atabecestat^154^: As a clinical nonselective BACE1 inhibitor, it reduced Aβ but failed to improve cognition. It had an adverse impact on liver enzymes, resulting in liver enzyme‐related side effects.

CNP520^155^: This is a BACE1 selective inhibitor that could reduce Aβ in the brain and CSF. Its efficacy is anticipated to be reported in 2024.

Semagacestat^156^: This is a γ‐enzyme inhibitor that could reduce Aβ production but induced side effects such as weight loss, increased incidence of skin cancer and infection, laboratory abnormalities including decreased levels of lymphocytes, T cells, immunoglobulins, albumin, and uric acid, and increased levels of eosinophils, monocytes, and cholesterol.

Tarenflurbil^157^: This is a γ‐enzyme inhibitor that failed to improve cognition and slightly increased the frequency of dizziness, anemia, and infections.

For a detailed description of three drugs targeting soluble Aβ (semagacestat, verubecestat, solanezumab, and crenezumab) and three targeting (bapineuzumab, gantenerumab, lecanemab, aducanumab, and donanemab).^4^ Panza et al.^5^ additionally described four BACE1 inhibitors, including Lanabecestat, Elenbecestat, Atabecestat, and CNP520.

For these clinical studies with rigorous designs, there were still many limitations. Therefore, Karran et al.^2^, ^4^ proposed that an ideal anti‐Aβ drug trial needs to determine the effective threshold and select appropriate patients. After further summarization, we propose that a good design should at least have the following characteristics: (1) The drug needs to have sufficient half‐life, BBB penetration ability, and ability to remove Aβ at a uniform rate; (2) the specificity of the drug needs special consideration to avoid possible immune inflammatory cascades and systemic reactions; (3) the selection of patients needs to be representative, and due to existing evidence that clinical typing and genotyping have a major impact on the prognosis of patients and Aβ pathological effects, it is also necessary to distinguish the subtypes of patients; and (4) it is necessary to find a specific treatment window period because different disease process stages correspond to different pathological changes, and the choice of the treatment window period will determine the efficacy in preserving neurological function. Furthermore, it is essential to recognize that preclinical studies typically utilize animal models, which may not fully represent human physiology. Transgenic animal models, commonly used in research, are developed under conditions of complete gene ablation, resulting in potential disparities between animal and human responses to drugs. Therefore, careful consideration of these differences is imperative when interpreting and extrapolating study results.

## 7 LABORATORY‐PROVEN TREATMENT

**Figure 2:**
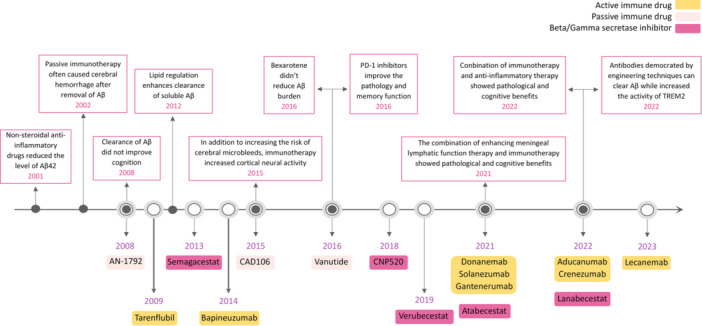
Development of representative clinical drugs and laboratory experiments of Aβ therapy. Aβ, amyloid‐β; PD‐1 inhibitors, programmed death‐1 inhibitors; TREM2, triggering receptor expressed on myeloid cells 2. [Color figure can be viewed at wileyonlinelibrary.com]

This section presents a comprehensive overview of the basic research status of Aβ treatments in animal and cell models (Figure 2).

### 7.1 Immunotherapy

After Demattos et al. initially injected a monoclonal antibody (mAb), m266, into mice, the Aβ burden in the mouse decreased. At the same time, the Aβ levels in the peripheral blood increased sharply, and the degree of this increase was related to the burden of Aβ in the brain.^158^ This was the first time that the role of monoclonal antibodies in the treatment of AD was verified, and it was found that the Aβ levels in the brain decreased by mAb were cleared into the peripheral blood through a certain pathway.

However, further studies have proved that the deposition of Aβ in cerebral blood vessels leads to loss of smooth muscle cells and damage to blood vessel walls in mice and humans. This mechanism induces cerebral hemorrhage as a common side effect caused by passive immunotherapy, and the area where cerebral hemorrhage occurs is highly correlated with the brain area affected by amyloid angiopathy (CAA).^159^ Therefore, this is also the most important reason for the failure of trials of drug developments. For example, Holmes et al.^132^ reported that there was no improvement in the cognition of mice after clearance of Aβ by injection of full‐length Aβ peptide, perhaps because the benefits of removal of Aβ and the side effects of immunotherapy counteracted each other. Therefore, we propose that supplementing anti‐Aβ therapy with anti‐inflammatory therapy and local immunosuppressive therapy or focusing on vascular protection may remove Aβ while reducing side effects. It should also be noted that, in addition to increasing the risk of cerebral microbleeds, antibody immunotherapy reduces Aβ while increasing cortical neural activity,^160^ leading to neurological damage.

If the side effects of mAb drugs are due to immune overactivity, what if we attempt to treat AD by suppressing the activity of the immune system? Baruch^161^ found that systemic immunosuppression may impair the ability of the brain to mount a protective, cell‐mediated immune response required for repair, and they reported improved pathology and memory in AD mice using programmed death‐1 (PD‐1) inhibitors, a type of cancer immunotherapy. The underlying mechanism is that immune checkpoint blockade against the PD‐1 pathway induces an interferon‐γ‐dependent systemic immune response, followed by the recruitment of monocyte‐derived macrophages to the brain, leading to the clearance of Aβ and improved cognitive performance. In addition, soluble TREM2 may also be a key indicator for clinical trial design and interpretation.^93^ Based on the effects of TREM2 and SYK on Aβ in vivo, in another study, an antibody against CLEC7A, a receptor that directly activates SYK, was designed to rescue microglia activation in mice expressing the TREM2R47H allele.^89^ Zhao et al.^162^ also developed an engineering method using antibodies to increase the activity of TREM2 by 100 times, thereby clearing Aβ in mice. Finally, new evidence shows that enhancing meningeal lymphatic function in combination with immunotherapy may lead to better clinical outcomes.^96^

### 7.2 Anti‐inflammatory therapy

Anti‐inflammatory therapy aims to reduce the level of inflammatory factors such as ILs in the brain and control the intensity of the inflammatory response, thereby alleviating inflammatory injury to the brain. The original study found that short‐term administration of ibuprofen significantly reduced the levels of Aβ_42_ in mice. After further in vitro experiments, it was found that the decrease of Aβ_42_ secretion was accompanied by an increase of Aβ_1‐38_ isomers. Therefore, two conclusions were drawn: (1) nonsteroidal anti‐inflammatory drugs (NSAIDs) can inhibit the production of Aβ_42_ and (2) NSAIDs may exert this inhibitory effect by changing the activity of γ‐secretase rather than cyclooxygenase (COX) enzyme activity.^163^

Mitochondrial defect is a key event in the pathogenesis of AD, since it damages nerve cells by altering microglia phagocytosis and triggering neuroinflammation. Aβ deposition can be inhibited by inducing microglia phagocytosis and antineuroinflammation; thus, cognitive deficits can be reversed.^164^ In addition to mitochondrial defects, astrocytes can also trigger transcriptional, morphological, and functional programming of microglia through programmed production of IL‐3, endowing them with an acute immune response program, enhanced motility, and the ability to accumulate and clear Aβ and tau aggregates, thereby limiting AD pathology and cognitive decline.^165^ In the latest study, it was found that, on the basis of Aβ antibody treatment, anti‐inflammatory treatment can significantly reduce synapse elimination and microbleeding in AD and CAA model mice, resulting in better behavioral outcomes.^166^

### 7.3 Lipid improvement

APOE plays a crucial role in lipid transport and maintenance of synaptic homeostasis. Therefore, AD‐related pathways may be regulated by increasing the amount of APOE lipidation. Oral administration of the retinoid X receptors agonist bexarotene to a mouse model of AD enhanced the clearance of soluble Aβ within hours in an APOE‐dependent manner.^167^ Within 72 h, the Aβ plaque area was reduced by more than 50%, and cholesterol transporter and high‐density lipoprotein levels were increased. Although bexarotene can induce a 25% increase in CSF APOE levels, it cannot reduce the Aβ burden in AD patients due to poor central penetration.^168^ Adeno‐associated virus‐mediated overexpression of APOE4 in a mouse model exacerbated synapse loss and Aβ deposition, whereas overexpression of APOE2 in the same model resulted in decreased Aβ levels in the brain.^169^ Consistently, after crossing knock‐in mice with APOE 2 expression with mice with APOE4 expression, the Aβ deposition of offspring was considerably reduced.^170^ Furthermore, overexpression of APOE4 increased the levels of hypolipidated APOE in the brain, whereas overexpression of APOE2 increased the lipidation of APOE.^171^ Therefore, the increased expression of APOE2, but not APOE4, may enable the alleviation of Aβ pathology. In addition, APOE deficiency may also be related to inflammation. Oxidized lipids in APOE‐deficient mice activate the classical complement cascade, leading to leukocyte infiltration of the choroid plexus.^172^ Notably, bexarotene stimulated rapid reversal of cognitive, social, and olfactory deficits, and improved neural function, and was effective for both early and slightly later stages. However, it takes 20 days for oral bexarotene to lower Aβ, which means that APOE therapy is more likely to be a long‐term chronic intervention, and this intervention is more likely to promote cognitive improvement by intervening in vascular risk factors (APOE is a common vascular risk factor). Does this also suggest that the vascular clearance pathway effectively controls Aβ levels and improves cognition?

Finally, some nanomedicines based on lipid receptors are being developed. LRP1 is a lipoprotein receptor that is downregulated in AD mice.^173^, ^174^ Tian et al. developed an LRP1‐targeted nanodrug with good BBB permeability based on a multivalent targeting strategy that can alleviate the deposition of Aβ in APP mice within 2 h. Since the mechanism of this drug is to improve the clearance system of Aβ, its immune induction and inflammatory response are mild.^175^, ^176^, ^177^

### 7.4 Others

Apart from the antibody‐based immunotherapy, anti‐inflammatory therapy, and lipid‐modulation strategies mentioned above, there are other methods that can regulate Aβ, including neuromodulation,^178^ antiaging therapy,^179^, ^180^ protein/cell activity modulation,^39^, ^181^, ^182^, ^183^, ^184^ gene regulation,^44^, ^185^, ^186^ and inorganic salt.^85^ A summary of these treatments is presented in Table 2.

**Table 2:** Brief summary of other treatments.

Treatment	Mechanism/method	Result	References
Gene editing	Knocked out LilrB2	The mice showed remission of AD‐like symptoms.	[185]
	CRISPR‐Cas9 amphiphilic nanocomplex for BACE1 gene editing in vivo neurons	Alleviated defects in mouse models of Alzheimer's disease.	[186]
	Knocked out Arc	Reduced the aβ load in AD transgenic mouse models.	[44]
Neuroregulation	Inhibited GABaergic neurons	Ameliorated Aβ‐dependent slow wave propagation damage.	[178]
Antiaging	Oral gavage with dasatinib and quercetin (Selleckchem)	Alleviated aging and cognitive deficits in Aβ‐related oligodendrocyte progenitors, but not astrocytes, microglia, or oligodendrocytes.	[180]
	Inhibited aging by insulin‐like molecules	Reduced toxicity mediated by Aβ aggregation	[179]
Regulation of protein or cell	Use chaperone proteins to produce small‐molecule inhibitors of amyloid aggregation.	Some inhibitors can interact with Aβ to reduce its toxicity.	[184]
	Inhibited α‐synuclein	Inhibited the deposition of Aβ.	[183]
	Inhibited ABAD–Aβ interaction specifically by an ABAD peptide.	Inhibited Aβ‐induced neuronal apoptosis and free radical generation.	[39]
	An ubiquitin hydrolase(Uch‐L1)	Reversed the Aβ‐induced decline in synaptic function and contextual memory.	[181]
	Inhibited the IGF pathway	Slowed the progression of Aβ.	[182]
Others	Therapeutic dose of lithium (a GSK‐3 inhibitor)	Interfered with APP cleavage in the gamma‐secretase step and blocks beta peptide production, but does not inhibit Notch processing, affect APP production, or change the structure of the C‐terminal or N‐terminal of presenin.	[85]

Abbreviations: ABAD, Abeta‐binding alcohol dehydrogenase; LilrB2, leukocyte immunoglobulin‐like receptor B2.

It should be noted that while the array of therapeutic strategies is promising in terms of alleviating the damage caused by Aβ, it also adds complexity to treatment methods. Therefore, researchers must carefully consider the selection of treatment strategies to ensure optimal outcomes.

## 8 CONCLUSION AND PROSPECT

Aβ research can be summarized into four categories: (1) direct observation of the structure of Aβ through advanced microscopic techniques; (2) exploration of the influence of genes, proteins, and pathways on Aβ in vitro or in model mice by means of genetics, cell biology, and biochemistry; (3) identification of the level and distribution characteristics of Aβ in the brain through clinical data, imaging techniques, and biochemical analysis techniques for clinical diagnosis or prognosis prediction; and (4) design of anti‐Aβ drug to inhibit generation of Aβ or improve the clearance of Aβ. Among these, studies on microglia, mitochondria, lysosomes, inflammatory factors, vascular factors, PET, APP, APOE, PS, tau, TREM2, monoclonal antibodies, and β/γ secretase inhibitors are the hottest topics.

While previous studies predominantly focused on changes of Aβ levels, contemporary research is increasingly focused on investigating the different distribution characteristics of Aβ as it is widely accepted that accumulation of Aβ is more pathogenic than the increase of Aβ.^107^ Understanding the distribution of Aβ not only sheds light on the progression of the disease but also has significant implications for therapeutic development. For example, in one‐third of mild to severe AD APOE4 noncarriers, Aβ is deposited in the cerebral cortex, suggesting that these patients may gain limited or no benefit from anti‐Aβ therapy.^117^ In order to further clarify the distribution of Aβ, new technical methods are constantly being developed. Ni et al.^187^ longitudinally assessed Aβ deposition in a transgenic mouse model by submillimeter‐scale invasive intravital microscopy combined with large‐field multifocal illumination fluorescence microscopy and panoramic volume multispectral photoacoustic tomography. Finally, they confirmed the specificity and regional distribution of Aβ with brain tissue sections.

However, Aβ positivity is not a specific manifestation in AD patients. Even in controls with normal cognition, MCI patients, and non‐AD neurodegenerative disease patients, the Aβ positivity rate was reported to be 26.3%, 65.9%, and 23.8%, respectively; however, the Aβ positivity rate was reported to be 100% in AD patients.^108^ This means that (1) Aβ can be used as a specific diagnostic biomarker with an acceptable rate of false positivity. (2) The accumulation of Aβ may reflect the degree of nerve damage, which also means that Aβ has a good relationship with natural progression of age and other neurological diseases. A question therefore arises: what is the relationship between Aβ and the manifestation of neurodegenerative changes? Mormino et al.^188^ specifically studied a group of SNAP individuals who were negative for Aβ (Aβ−) but positive for neurodegeneration and found that CN individuals with SNAP did not show increased pathological levels or increased risk of cognitive decline. Another study conducted on CN individuals also suggested that hippocampal volume or cortical glucose metabolism from AD‐vulnerable regions did not have a significantly different risk of cognitive decline, and the occurrence of neurodegeneration requires the synergy of Aβ.^189^ These results suggested that the emergence of Aβ and the change of the neural structure potentially occur as two autonomous processes, and neurodegeneration can only occur when they work together.

Based on previous studies on Aβ, the following conclusions are delineated: (1) Aβ accumulation is mainly due to impaired Aβ clearance caused by an impaired clearance system in the brain rather than the overgeneration of Aβ^10^, ^15^, ^94^; (2) a comprehensive therapy that simultaneously regulates Aβ pathological markers, inhibits neuroinflammation, and regulates lipids may have greater benefits^96^; and (3) the side effects of antibody therapy are mainly related to immune inflammatory storms.^132^, ^159^, ^160^ Before the official approval of Lecanemab, “whether Aβ removal is beneficial to cognitive improvement” was unclear to many developers. A systematic review that combined 19 randomized‐controlled trials (RCTs) unveiled that drugs that increased Aβ clearance improved cognitive function, whereas those that decreased Aβ production did not.^190^ However, an instrumental variable meta‐analysis ^191^ published in 2021 pooling 14 RCTs used a rigorous and efficient approach to test the Aβ hypothesis. The authors concluded that reductions in Aβ levels were unlikely to slow cognitive decline. Yet, Pang et al.^192^ reproduced this study, rectified input data discrepancies, and supplemented overlooked trial data that were omitted from the original publication, subsequently revealing consistent and statistically significant beneficial effects of Aβ reduction on three commonly used clinical outcome measures in AD.

Recently, in addition to traditional drug therapy, there have been many emerging therapies for AD, among which the most promising are nanomedical therapy^193^ and intestinal microbial therapy.^49^, ^194^, ^195^ It is widely acknowledged that LRP1 is critical for regulating BBB in the CNS and is associated with Aβ accumulation, neuroinflammation, cerebral blood flow and metabolism, changes in synaptic transmission, and neuronal damage.^196^ Tian et al. found that targeting LRP1 by multivalent targeting nanotechnology may potentially lead to active transcytosis.^176^, ^197^ In AD, the decrease of expression of LRP1 in microvessels exacerbated disease progression.^198^, ^199^, ^200^ Some nanoparticles (NPs) have successfully reduced Aβ accumulation by upregulating LRP1 expression on BBB^201^ and improved AD‐like pathological manifestations in APP/PS1 mice.^202^ Multivalent targeting technology is a method in the field of nanotechnology to target specific molecules by designing receptor‐specific ligands. The main principle is to allow multiple ligand–receptor pairs to bind simultaneously to improve binding selectivity and affinity, while prolonging the duration of the interaction.^203^ Ligand‐functionalized NPs can selectively bind specific cellular receptors and can undergo receptor‐mediated endocytosis, leading to internalization of ligand–receptor complexes and subsequent intracellular delivery of NPs and therapeutic agents. This approach, compared with conventional drugs, presents several advantages, including (1) increased drug biodistribution by preventing opsonization and hepatic clearance; (2) macromolecular stability to physiological‐like conditions; (3) phenotype targeting to avoid off‐target side effects; and (4) good central concentration that can improve drug efficacy by reducing the effective dose of treatment, to reduce or eliminate drug side effects. Interestingly, a recent study found that acousto‐optic frequency stimulation can have a good therapeutic effect on AD; this has passed phase 2 clinical trials and is also worthy of attention.^204^ Moreover, the influence of aging, living environment, and lifestyle on patients should also be weighed on the basis of ensuring the feasibility and interpretability of the experiment. Recent studies have also noted that physical exercise independently attenuated the inverse association of Aβ burden with cognitive decline and neurodegeneration in asymptomatic individuals.^205^ The therapeutic potential of environmental enrichment has been substantiated by its capacity to significantly reduce Aβ deposition levels in the brain of transgenic mice, as well as to selectively upregulate transcript levels of genetic codes related to learning and memory, angiogenesis, neurogenesis, cell survival pathways, Aβ sequestration, and prostaglandin synthesis.^206^ Above all, it is imperative to underscore that although Lecanemab is the first drug to be approved by the FDA, improvement of cognition is a multifactorial subject. More interdisciplinary research technologies and clinical considerations need to be introduced into neurodegenerative disease research.

Finally, the relationship between brain structure and function is the main focus of study in neurodegenerative diseases. Recently, Pang et al.^207^ proved that the geometric structure of brain plays a more important role in the function of the brain than neural connection, as was traditionally believed. This novel perspective may open up a new horizon for AD research, potentially enabling a more three‐dimensional comprehension of brain dynamics.

## AUTHOR CONTRIBUTIONS

All authors have read and approved this paper. Yifan Yu drafted, developed, and revised this paper, and conceived the figures. Shilong Yu drafted this paper and organized the tables for this paper. Giuseppe Battaglia and Xiaohe Tian revised this paper. All authors have read and approved the final manuscript.

## CONFLICT OF INTEREST STATEMENT

Prof. Xiaohe Tian and Giuseppe Battaglia are the associated editors of Ibrain. They have fully revealed these interests, and have worked out a plan for approval to manage any potential conflicts caused by their participation. Other authors have no conflict of interest to disclose. Xiao‐He Tian and Giuseppe Battaglia were excluded from all the editorial decisions.

## ETHICS STATEMENT

The authors have nothing to report.

## Data Availability

The authors have nothing to report.
